# Protective Effect of Selenium-Enriched Ricegrass Juice against Cadmium-Induced Toxicity and DNA Damage in HEK293 Kidney Cells

**DOI:** 10.3390/foods7060081

**Published:** 2018-05-28

**Authors:** Rattanamanee Chomchan, Sunisa Siripongvutikorn, Pattaravan Maliyam, Bandhita Saibandith, Panupong Puttarak

**Affiliations:** 1 Interdisciplinary Graduate School of Nutraceutical and Functional Food, Prince of Songkla University, Hat-Yai, Songkhla 90112, Thailand; pui_galz@hotmail.com (R.C.); bandhita.s@psu.ac.th (B.S.); 2 Department of Food Technology, Faculty of Agro-Industry, Prince of Songkla University, Hat-Yai, Songkhla 90112, Thailand; sunisa.s@psu.ac.th; 3 Department of Pharmacognosy and Pharmaceutical Botany, Faculty of Pharmaceutical Sciences, Prince of Songkla University, Hat-Yai, Songkhla 90112, Thailand; Tak_kypoko@hotmail.com; 4 Phytomedicine and Pharmaceutical Biotechnology Excellence Center (PPBEC), Prince of Songkla University, Hat-Yai, Songkhla 90112, Thailand

**Keywords:** anti-cadmium toxicity, comet assay, DNA protective, HEK293 cells, ricegrass juice, selenium enrichment

## Abstract

Cadmium (Cd) contamination in food is a problem endangering human health. Cd detoxication is an interesting topic particularly using food which provides no side effects. Ricegrass juice is a squeezed juice from young rice leaves which is introduced as a functional drink rich in polyphenol components. Se-enrichment into ricegrass is initiated to provide extra advantages of their functional properties. The protective role of ricegrass juice (RG) and Se-enriched ricegrass juice (Se-RG) against Cd toxicity during pre-, co- and post-treatment on HEK293 kidney cells were investigated. Results confirmed that RG and Se-RG had very low toxicity for kidney cells. Both extracts showed a protective role during pre-treatment and co-treatment against Cd toxicity by exerting a reduction in malondialdehyde (MDA) content and the percentage of DNA damage in tail and tail length of the comets over the Cd-treated cells. However, the Se-RG indicated additional benefits in all properties over RG. High Se content in Se-RG resulted in more protective effects of the regular ricegrass juice. In summary, this study provides clear evidence that Se-enriched ricegrass juice has potential to be developed as a functional food to protect the human body from Cd contamination via the reduction of oxidative stress and DNA damage.

## 1. Introduction

Currently, daily food consumption could lead to the unexpected exposure of contaminated compounds in the human body. Heavy metals, known as harmful agents, enter the food chain excessively via industrial operations, mining, sewage sludge and waste disposal from households into agricultural lands and water resources [1]. Accumulation of heavy metals in the environment has been reported as increasing substantially over the past decades [2]. Due to the highly soluble ability of cadmium (Cd) compounds as compared to other metals, Cd is readily taken up by plants resulting in food and feed accumulation. Cd contamination from the environment is a subject of serious health complications affecting cellular organelles and components such as cell membrane, mitochondrial, lysosome as well as genetic DNA [3]. The kidney is a critical target organ where Cd is predominantly bioaccumulated. The exposed level of Cd can cause chronic difficulties, thus leading to damage of kidney filtering mechanisms, kidney dysfunction, liver damage as well as damage to skeletal, reproductive and respiratory systems [4,5]. The mechanism of Cd toxicity is related to its interaction with carboxyl and thiol groups of protein which generate the production of reactive oxygen species (ROS) such as superoxide ions, hydrogen peroxides, and hydroxyl radicals, and therefore inducing oxidative stress and DNA damage by initiation of lipid peroxidation [6].

Recently, several studies have been reported that antioxidant molecules have protective effects against renal and hepatic cadmium toxicity via a function of free radical scavenging [7]. Plants are the foremost source of natural antioxidant molecules such as vitamin C, vitamin E, polyphenols and some minerals like Se and Zn [8], thus they can be hypothesized as effective anti-cadmium toxicity materials. The discovery of functional plant food rich in antioxidant compounds is now being considered. Sprouts or young plants of cereals, grains or legumes are currently of interest since plants at the beginning of the growing stage are associated with large amounts of quality bioactive compounds and antioxidant molecules like polyphenols. Ricegrass is a brand-new sprout which was recently introduced as a substitute for wheatgrass, particularly in tropical areas, as a low-cost ingredient. It is rich in polyphenol compounds and has been previously investigated for its ability to scavenge free radicals in vitro effectively [9].

It was proposed that the human body attempts to reduce heavy metals toxicity via some antioxidant mechanisms such as metal chelation or degradation of free radicals [10]. Selenium (Se) has been stated as a cofactor of antioxidant enzymes and can be used as antidote agent for mercury (Hg), cadmium (Cd) and silver (Ag) [11]. The enrichment of Se into plants has been studied worldwide to increase the level of Se content and may possibly propose an extra role for the biological properties in plant foods, although concern must be given to appropriate forms and concentrations of Se [12]. Therefore, the objective of this study was to identify the specific phenolic types of polyphenol in ricegrass juice extract (RG) and Se-rich ricegrass juice extract (Se-RG) and to investigate the effect of them on in vitro anti-cadmium toxicity in HEK293 (human embryonic kidney cells kidney cells), lipid peroxidation and DNA protective properties.

## 2. Materials and Methods

### 2.1. Reagents

Di-sodium ethylenediaminetetraacetic acid (EDTA-Na_2_), malondialdehyde (MDA), 2-thiobarbituric acid (TBA) and 3-(4,5-dimethylthiazol-2-yl)-2,5-diphenyltetrazolium bromide (MTT) were acquired from Sigma Aldrich Co. (St. Louis, MO, USA). SYBR gold nucleic acid stain and trichloroacetic acid (TCA) were purchased from Thermo Fisher Scientific Co. (San Jose, CA, USA). Reagents and media for the cell line included a trypan blue dye, trypsin-EDTA, fetal bovine serum (FBS), penicillin-streptomycin and Dulbecco’s Modified Eagle Medium (DMEM) were purchased from Gibco BRL, Life Technologies Inc. (Rockville, MD, USA). Low melting point agarose (LMA), dimethyl sulfoxide (DMSO) and Triton-X was purchased from Amresco Inc. (Solon, OH, USA).

### 2.2. Plant Materials

*Oryza sativa* L. cv. Chainat 1 obtained from the Phatthalung Rice Research Center, Phatthalung, Thailand was used in this study. Se in the form of sodium selenite at range 40 mg Se/L was used to produce Se rich ricegrass as the best enrichment condition according to previous work [13]. Regular young ricegrass and Se-rich ricegrass was grown for 8 d. After harvesting, both grasses were aqueous extracted and the juices were lyophilized into powder. Ricegrass juice extract (RG) and Se-enriched ricegrass juice extract (Se-RG) were determined for the total Se content using induced coupled plasma optical emission spectroscopy (ICP-OES) and the contents were reported as 1.3 and 59.8 µg/g of extract, respectively.

### 2.3. Polyphenols Identification

RG and Se-RG were investigated for the major compounds using the reversed-phase ultra-high-performance liquid chromatography–electron spray ionization–mass detector (UHPLC–ESI–MS) since only restricted data has been stated earlier on the specific types of polyphenols found in the aqueous extract of young ricegrass. Polyphenols in RG and Se-RG were identified using a Thermo Scientific (Dionex Softron GmbH., Germering, Germany) Ultimate 3000 UHPLC system equipped with diode array absorbance detector, electron spray ionization and linear ion trap (LTQ XL) mass detector (DAD-ESI-MS). 10 mg/mL of extracts were dissolved in HPLC water and filtered with a sterile syringe filter of 0.45 µm. Purosper STAR (250 mm × 4.6 mm) with LiChrocart, Reverse Phase-18 column end-capped with 5 µm diameter particles (Merck, Darmstadt, Germany) was used as the stationary phase. H_2_O containing 0.5% formic acid (solvent A) and acetonitrile (solvent B) were selected as the mobile phase. The gradient condition was run according to Table 1 and followed by washing with 100% methanol for 15 min and re-equilibration. The flow rate was 0.8 mL/min, column temperature was 40 °C with the injection volume of 20 μL. The MS parameters were as follows for both the negative and positive mode: heater temperature: 250 °C; capillary temperature: 330 °C; sheath gas flow: 50 arbitrary units; auxiliary gas flow: 10 arbitrary units. The mass data for the molecular ions were processed with Thermo XcaliburTM software version 2.2.44 (Thermo Scientific., Hemel Hempstead, UK). The peaks were examined based on the ultraviolet (UV) spectra and mass ion compared to the existing literature.

### 2.4. Cell Culture Model

HEK293, human embryonic kidney cells, were purchased from the American Type Culture Collection (Manassas, VA, USA). Dulbecco’s Modified Eagle Medium (DMEM) supplemented with 1.5% sodium bicarbonate, 10% fetal bovine serum (FBS), 1% penicillin-streptomycin was used for the maintenance of cells at 37 °C, 5% CO_2_, in a fully humidified incubator. Phosphate buffer saline (PBS) at pH 7.2 was used to wash the cells through the experiment.

### 2.5. Cell Viability Assay

Functional food products which can be claimed as safe need to be confirmed on their viability effect of mammalian cells. The experiment was operated on human embryonic kidney cells, HEK293, to define the dose of extracts indicated as safe to the cells to be used for the anti-cadmium toxicity test. The MTT assay was used to determine the cell viability. Cells grown at 80–90% confluent were harvested with 0.25% trypsin–EDTA and suspended in a fresh medium. Cell counts were measured using a standard haemocytometer based trypan blue cell counting technique [14]. HEK293 cells at the density of 1 × 10^6^ cells/mL were seeded in 96-well tissue culture plates and allowed to adhere for 24 h. After the cells were washed with PBS (pH 7.2), the media was mixed with a various concentration of the extracts (250–10,000 µg/mL), then applied to the cells followed by 24 h incubation. After 24 h, the cell viability was evaluated by adding 20 µL of MTT solution and 2 h incubation. Afterward, the MTT solution was removed, and 100 µL of 0.04 N HCl in isopropanol was added to dissolve the formazan crystals. Absorbances were recorded at 570 nm using a microplate reader. The percentage of cell viability was calculated with Equation (1);

% Cell viability = (Absorbance of sample/Absorbance of control) × 100(1)

### 2.6. Anti-Cadmium Toxicity Properties

To examine the anti-cadmium toxicity properties of RG and Se-RG in HEK293 cells, initially, the half maximal cytotoxicity concentration (CC_50_) of CdCl_2_ was investigated to be used as the established dose to induce toxicity to the cells. After seeding HEK293 cells into 96-well plates at a density of 1 × 10^6^ cells/mL, the cells were left to attach for 24 h before being treated with either CdCl_2_ or extracts. The experiment was divided into three groups separated by different time order for treating the cells with extracts. The first treatment group was the extracts’ pre-treatment; the ideal substances that can reduce toxicity from this treatment could represent the role of the protective substances. Secondly, the group of co-treatment was examined to check if the extracts could provide a protective role to cells while it directly reacted to Cd. Lastly, the post-treatment group was designed to indicate the role of the extracts as therapeutic agents. The detail of each group is briefly indicated in Table 2. The extract was fixed to have a contact time of 24 h on cells in all treatments. The percentage of cell viability was detected by MTT cytotoxicity assay and calculated as Equation (1). The morphology of the cells in each treatment was also observed and captured using a microscope.

### 2.7. Determination of Lipid Peroxidation

TBARS (thiobarbituric acid reactive substances) assay was used to determine the level of lipid peroxidation. The endogenous cellular fluid was extracted according to the modified method of Du, et al. [15]. Cells were harvested with 0.25% trypsin-EDTA and followed by centrifugation at 1000× *g* for 10 min. Cell pellets were washed with cold PBS until clean and re-suspended in 1 mL of cold PBS. Cells were lysed using a probe-type sonicator (Vibra-Cell, Sonics and Materials Inc., Newtown, CT, USA) by pulsing at 15 s on and 10 s off for 5 cycles on ice. The cell extracts were centrifuged at 10,000× *g* (4 °C) to discard the cell debris while supernatants were used for the determination of MDA content and protein levels. Protein content was examined using bovine serum albumin (BSA) as standard [16]. The modified method of Chen, et al*.* [17] was used to determine the MDA content. 1 mL of cellular extracts were mixed with 4 mL of 20% TCA containing 0.8% of TBA (*w*/*v*). The mixtures were heated at 95 °C for 60 min, then cooled in ice and centrifuged at 3000× *g* for 10 min. The absorbance was measured at 532 nm. The amount of MDA–TBA red complexes were compared to an external standard of MDA. The amount of TBARS was expressed as nmol MDA/mg protein.

### 2.8. DNA Protective Properties Using Comet Assay

DNA damage can be induced by exogenous agents such as heavy metals, polycyclic aromatic hydrocarbon from pollution, endogenous chemical genotoxic agents such as reactive oxygen species (ROS) and natural chemical reactions [18]. The damage to the cellular genome can generate errors in the transcription of DNA and protein translation which impair signaling and the cellular function and could result in the development of diseases [19]. The alkaline single cell gel electrophoresis assay or comet assay was used to evaluate the DNA damage [20]. The DNA protective properties of the RG and Se-RG extracts on HEK293 cells towards the exposure to CdCl_2_ were investigated. Briefly, HEK293 cells were seeded at 1 × 10^6^ cells/mL in 12-well plates and incubated at 37 °C for 24 h. The cells were treated with the following condition stated above (Table 2). Then, cells were harvested and fixed into slides which had been covered with 150 μL of 1.5% LMA as the first layer. After solidification, 20 μL of freshly prepared cell suspension with 180 μL of 0.5% LMA (ratio 1:10) was rapidly mixed by pipetting, and 80 μL of the mixture was loaded as the second layer. Then, 70 μL of 1% LMA was added on to the cell layer as the third layer. Once the gel was solidified, the slides were placed in a chilled lysis buffer containing 2.5 M NaCl, 100 mM EDTA, 100 mM Tris–HCl at pH 10 and 1% DMSO, 1% Triton X-100 for at least 2 h at 4 °C. The slides were then removed and placed in a comet assay tank (Model CSL-COM20, Cleaver Scientific, Rugby, Warwickshire, UK) filled with freshly prepared alkaline buffer at 4 °C (300 mM NaOH, 1 mM Na_2_EDTA, pH ≥ 13) for 15 min to unwind the DNA, and electrophoresis was carried out at 25 V and 300 mA for 45 min. Afterward, the slides were rinsed with deionized water and neutralized gently with 0.4 M Tris–HCl buffer, pH 7.5 for 5 min. Finally, the slides were soaked in ethanol for 5 min and left at room temperature until they were completely dried. The cellular DNA was stained using SYBR gold nucleic acid stain in the dark for 20 min and visualized using a fluorescent microscope (Eclipse 80i, Nikon, Tokyo, Japan). The comet images (45–60 cells/slide) were captured and analyzed. The quantification of the DNA strand breaks was done using CometScore 2.0.0.38 software (Tritek Corp., Sumerduck, VA, USA). The % DNA in tail and tail length were obtained.

### 2.9. Statistical Analysis

Completely randomized design (CRD) was used throughout the study. All experimental data were presented as the mean ± standard deviation (SD) of three replications. Means were analyzed using analysis of variance (ANOVA). The significant differences among means were determined by Tukey’s test (*p* < 0.05) using SPSS for Windows (SPSS Inc, Chicago, IL, USA).

## 3. Results and Discussion

### 3.1. Polyphenols Identification Using Ultra High-Performance Liquid Chromatography–Electron Spray Ionization–Mass Detector (UHPLC–ESI–MS)

Results of polyphenols identification showed that RG and Se-RG contain similar types of identified compounds. Six major compounds comprising more than 70% of relative content were tentatively identified (Table 3) where the molecular weight and electrospray ionization mass spectrometry of detected compounds were reported. Phenolic glycoside was detected and defined as 1-*O*-sinapoyl-β-d-glucose. The ion found with the *m*/*z* of 367 was defined as 3-*O*-feruloyl quinic acid due to the presence of a ferulic acid fragment at *m*/*z* 193. The largest compounds in RG were identified as a group of flavone glycosides including chrysoeriol arabinosyl arabinoside, tricin, swertisin and tricin-7-*O*-β-d-glucopyranoside which has been earlier reported as having been found in the leaves of *Oryza sativa* and *Triticum aestivum* (wheat) [21,22].

### 3.2. Cytotoxicity of Ricegrass Juice Extract (RG) and Se-Rich Ricegrass Juice Extract (Se-RG)

**Figure 1:**
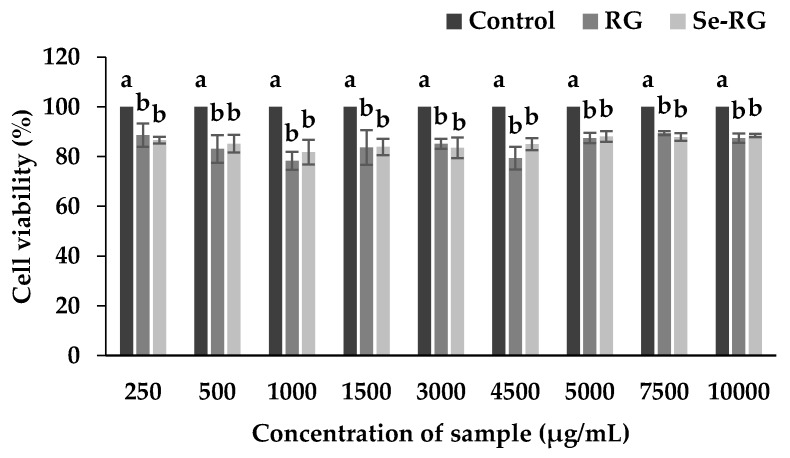
Effect of different concentrations of RG and Se-RG on the cell viability of HEK293 kidney cells. Data are means ± standard deviation (SD). Different letters indicated significant differences between treatment in the similar concentration of extracts (*p* < 0.05) in Tukey’s significant differences test.

The dose of extracts indicated as safe to cells was used for the anti-cadmium toxicity test. Figure 1 revealed that the cell number of HEK293 slightly decreases while treated with both RG and Se-RG and remained constant while the concentration of the extracts was increased up to the dose of 10,000 µg/mL. Se-RG extracts displayed no significantly different effect on the reduction of cell numbers compared to the RG (*p* < 0.05). There was a minor reduction in cell number, however, the cells remained higher than 80% of the control which was indicated as the acceptable range classified as safe [26]. Although the alteration of cell morphology after treatment with the extracts has been detected, it was regarded as a result of the sensitivity of cells when they had encountered the foreign matter and attempted to adapt to the new environment. Therefore, from this result it can be assumed that both extracts had no or low toxicity to the kidney cells and the dose of both extracts at the highest concentration (10,000 µg/mL) could be used for the next experiment.

### 3.3. Anti-Cd Toxicity of RG and Se-RG and Effect on Lipid Peroxidation

**Figure 2:**
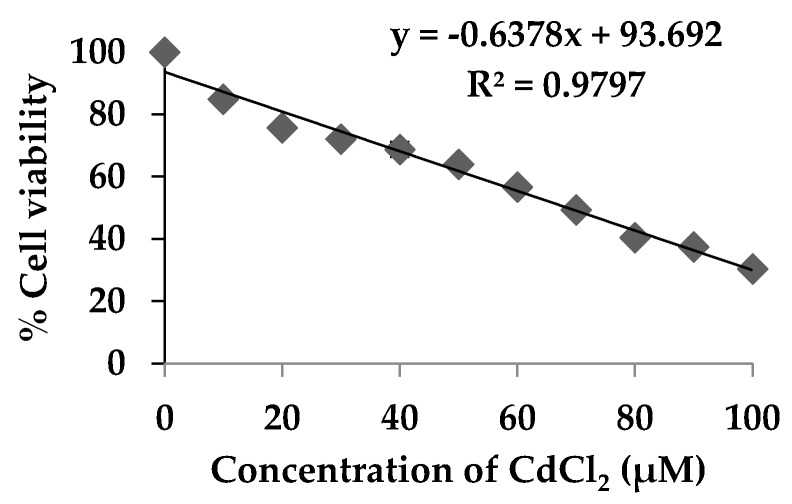
Effect of different concentrations of CdCl_2_ on the cell viability of HEK293 kidney cells. Data are means ± standard deviation (SD).

The kidney is the critical organ affected by chronic Cd exposure and toxicity. Cd accumulates in the kidney because of its preferential uptake by the receptor in the renal proximal tubule and accumulates in the human kidney for a relatively long time, from 20 to 30 years [27]. The exposed level of Cd can cause chronic difficulties, thus leading to damage of kidney filtering mechanisms and kidney dysfunction [4]. HEK293 cells, human embryonic kidney cells, were used as a demonstrative model to examine the effect of CdCl_2_ toxicity and the protective role of extracts against CdCl_2_. The level of CdCl_2_ which could be used to induce the cytotoxicity was examined. Figure 2 showed that the viability of HEK293 cells was significantly decreased (*p* < 0.05) while exposed to higher level of CdCl_2_. Cd is not able to generate radical itself but the toxicity was related to the generation of reactive oxygen species (ROS) such as superoxide ions, hydrogen peroxides and hydroxyls radicals and therefore induced oxidative stress and DNA damage by initiation of the lipid peroxidation [6].The estimated half maximal concentration (CC_50_) dose of CdCl_2_ in HEK293 was indicated as 68.5 µmol/L and this level could be used as a suitable dose for the evaluation of anti-cadmium toxicity properties of the RG and Se-RG.

**Figure 3:**
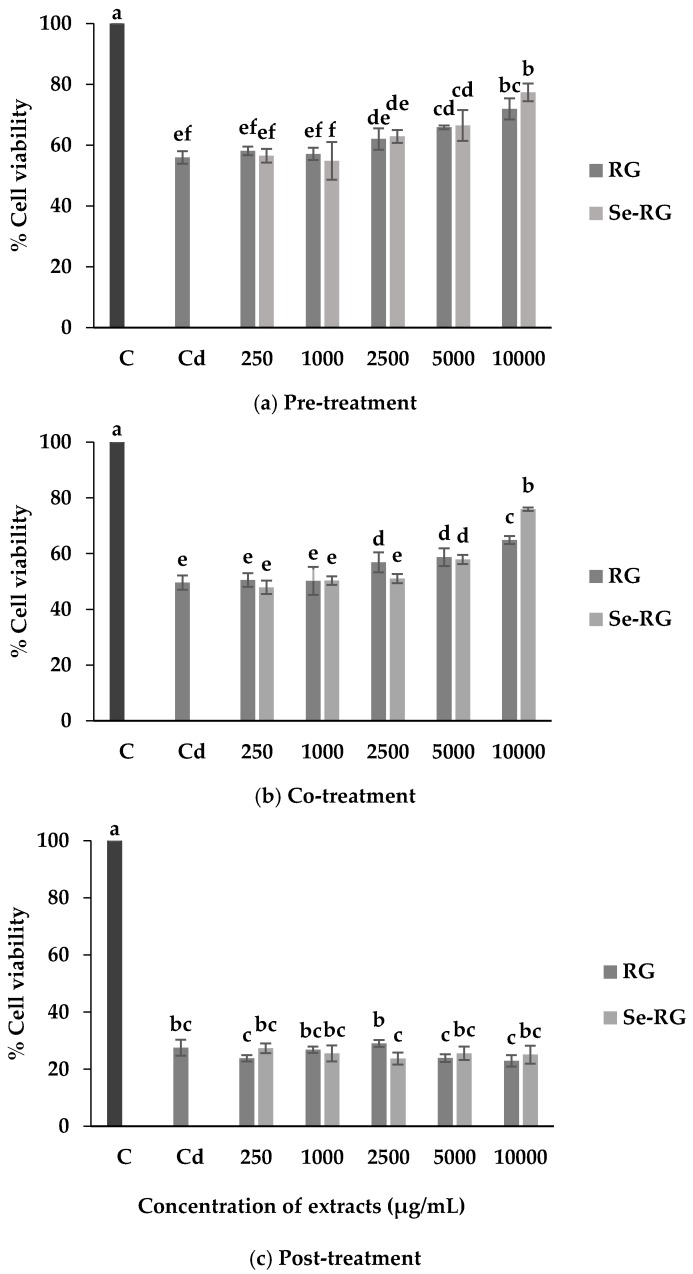
Anti-Cd toxicity properties of RG and Se-RG on HEK293 kidney cells while exposed to CdCl_2_ at different time orders of treating the extracts compared to the control and Cd-treated (**a**) pre-incubation (**b**) co-incubation (**c**) post-incubation. C means control; Cd means CdCl_2_ at CC_50_ level (68.50 µg/mL). The cell viability was expressed as a percentage of control. Data are means ± standard deviation (SD). Different letters indicated significant differences (*p* < 0.05) in Tukey’s significant differences test.

The experiment on anti-cadmium toxicity properties was designed to assess the effect of RG and Se-RG against CdCl_2_ exposure on the cell viability and lipid peroxidation at different time orders, as each substance may alleviate the toxicity from Cd induction differently [10]. Results reveal that RG and Se-RG significantly increased (*p* < 0.05) the percentage of cell viability during pre-treatment and co-treatment but not during post-treatment with Cd compared to the cells treated with Cd at CC_50_ level alone (Figure 3).

**Figure 4:**
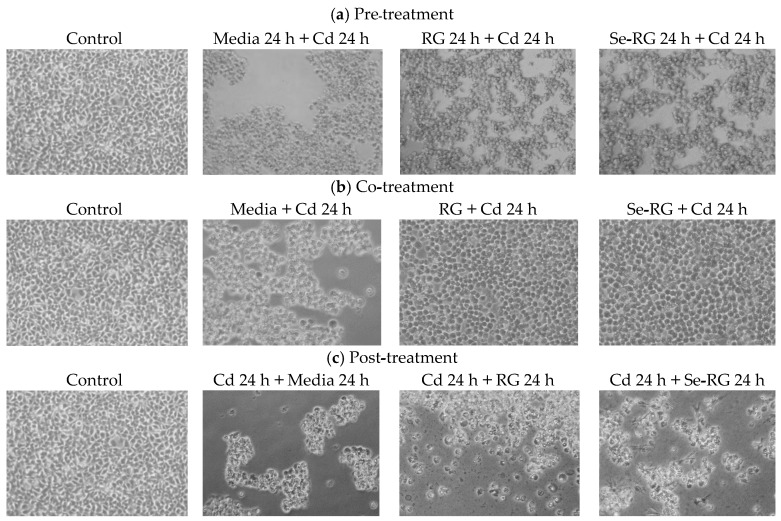
Morphology of cells of HEK293 kidney cells while incubated the cells with RG/Se-RG and CdCl_2_ at different time order of treating the extracts (**a**) pre-incubation (**b**) co-incubation and (**c**) post-incubation. C means control; Cd means CdCl_2_ at CC_50_ level (68.50 µg/mL).

**Figure 5:**
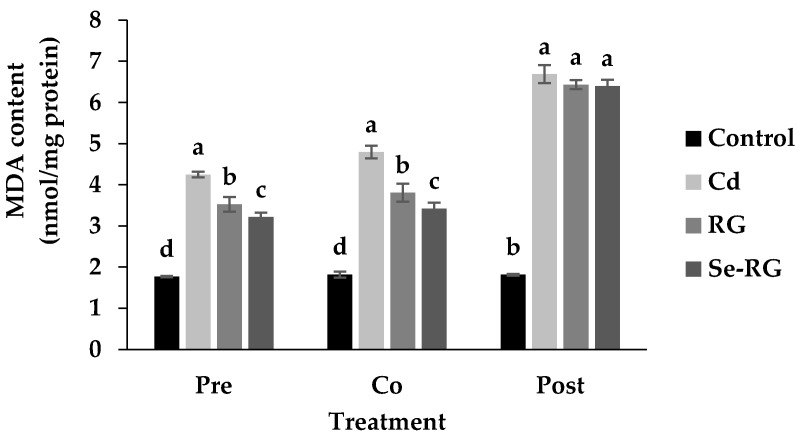
Level of malondialdehyde (MDA) in HEK293 cells treated with RG and Se-RG at the level 10,000 µg/mL compared to the control and Cd-treated ones. Data are means ± standard deviation (SD). Different letters indicated significant differences in the similar group of treatment (*p* < 0.05) in Tukey’s significant differences test.

The highest concentration of both extracts (10,000 µg/mL) exerted the highest ability to protect HEK293 cells against Cd toxicity. Thus, the pathological evaluation of cell morphology treated with the extracts at this concentration was observed as shown in Figure 4. The morphological changes while treating the cells with Cd were detected. A majority of the cells were broken and floated into the media while the rest were weakened and lost their cell structure. Although the cells in the condition of extracts pre-treatment illustrated some lost and unusual cells morphology, the cells remained strengthened in their frame similarly to web shape. The changes of cells in co-treatment conditions were also detected as they were swollen and changed to a circle-like shape, but they preserved their structure. These data suggested that pre-treatment and co-treatment of RG and Se-RG with Cd could improve the Cd-induced pathological damage of kidney cells better than the Cd-treated and can potentially protect against kidney cell damage. Living organisms contain lipid as the main structure of cellular membranes. Cd could induce the damaging effects to the cells from the lipid peroxidation process [28]. Therefore, the extent of lipid peroxidation by-products produced like malondialdehyde (MDA) can imitate the extent of cells oxidative damage initiated by Cd [29]. TBARS assay is a well-established method use as an index of lipid peroxidation and lipid hydroperoxides. When the cells exposed to Cd, the MDA content was markedly increased, thus suggesting the increased in oxidative stress of kidney cells (Figure 5). However, outcomes indicated that during pre-treatment and co-treatment of both RG and Se-RG, the level of MDA in HEK293 was significantly reduced compared to Cd-treated cells (*p* < 0.05).

The role of phenolic compounds in the extracts was considered as having the major effects on the protective role against Cd-induced damage. It could be explained that the extracts rich in polyphenols compounds possess the inhibition of lipid peroxidation chain reaction by stabilizing the hydroxyl radicals and lipid peroxyl radicals, thereby lowering the extent of oxidative damage to the lipid cell membrane and lower level of MDA [30,31]. Moreover, phenolic compounds as antioxidant molecules could propose the role of upregulating the antioxidant protection system by stimulating the production of antioxidant enzymes including super oxide dismutase (SOD), catalase (CAT), and glutathione peroxidase (GPx). As a result, strengthening the immunity and lowering the damage caused by Cd during pre-treatment and co-treatment to the cells [10]. RG and Se-RG contained abundant polyphenols such as flavone glycosides. Therefore, the protective role of the extracts could be related mainly to these groups of compounds. Similar results also indicated the protective effect of bioflavonoids, for example, quercetin against Cd-induced oxidative stress-related renal dysfunction in rats by attenuating the Cd-induced biochemical alterations in serum, urine and tissue pathological changes via a decrease in lipid peroxidation rate [32]. Flavonoid, namely catechin from green tea, has also been proved to protect against bone metabolic disorders in cadmium-poisoned rats [33]. While focusing on the effect of high Se, Se-RG extracts revealed marginally higher protective properties against Cd toxicity over the RG in pre-treatment and co-treatment conditions. Se was used as antidote agent to a range of heavy metal toxicities including Cd, Hg, and Ag [11]. Generally, studies have indicated the beneficial effect of Se on antioxidant status and lipid peroxidation when pre-exposed and co-exposed to Cd [34,35,36]. Lipid peroxidation occurred because of Cd exposure; moreover, a significant decrease in the antioxidant composition factors, such as glutathione (GSH) levels, the activities of glutathione peroxidase (GPx) and thioredoxin reductase (TrxR), was also stated [34]. Se compounds have been generally known as a major cofactor of GPx and TrxR, thus, Se could logically promote the greater level of these antioxidant enzymes activity and play a role in managing the radicals occurring in the cells. Se could also present protective effects on mitochondria dysfunction by blocking the ROS generation, a possible inhibition of Cd-induced mitochondrial membrane collapse [37].

### 3.4. DNA Protective Properties

**Figure 6:**
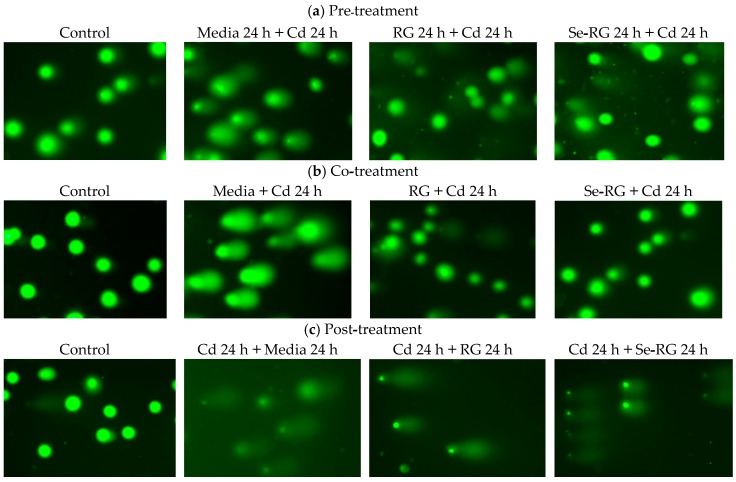
DNA protective effect of RG and Se-RG against CdCl_2_ induced DNA break in HEK293 kidney cells while incubated the cells with RG/Se-RG and CdCl_2_ at different time order of treating the extracts evaluated by comet assay (**a**) pre-incubation (**b**) co-incubation and (**c**) post-incubation. C means blank control; Cd means CdCl_2_ at CC_50_ level (68.50 µg/mL).

**Figure 7:**
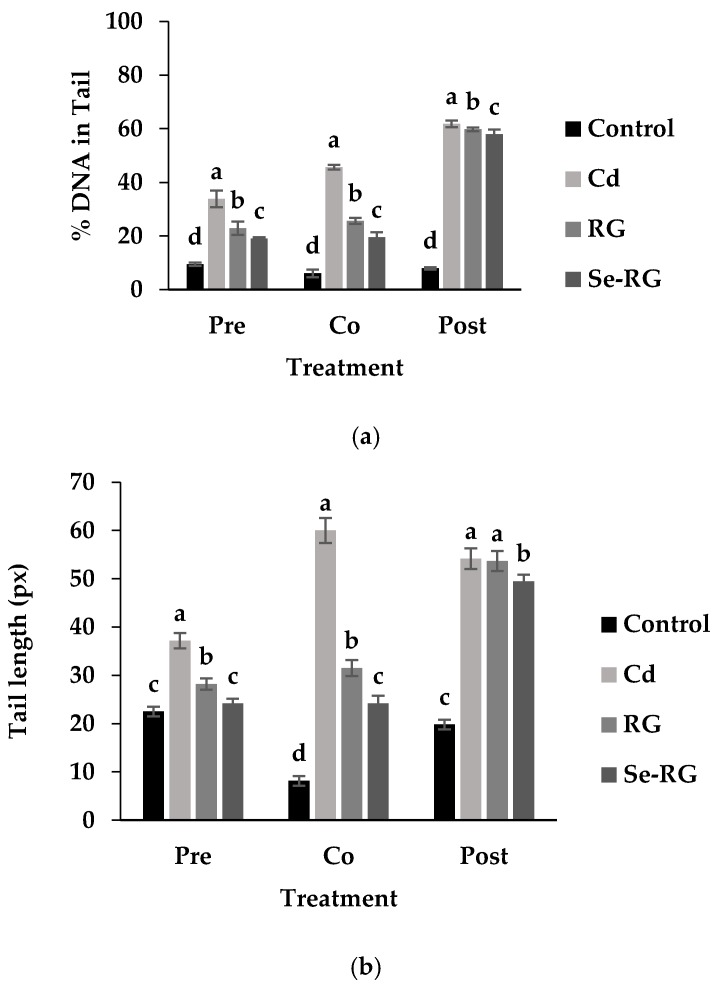
Assessment of DNA damage parameters in HEK293 cells treated with RG and Se-RG at the level 10,000 µg/mL compared to the control and Cd-treated ones evaluated using the CometScore software. (**a**) changes in % DNA in tail; (**b**) changes in tail length. Data are means ± standard deviation (SD). Different letters indicated significant differences (*p* < 0.05) in Tukey’s significant differences test.

Comet assay or single cell gel electrophoresis is a standard rapid method for detecting DNA damage in individual cells. The percentage of DNA in tail and tail length was analyzed as the measure of primary DNA damage [24]. Therefore, comet assay is useful for this study to evaluate the DNA protective role of RG and Se-RG extracts against Cd. The highest concentration of both was used (10,000 µg/mL) as they protect the highest number of percent cell viability. Figure 6 illustrates the capture of comet cells of each treatment during pre-treatment, co-treatment, and post-treatment of RG and Se-RG compared to the Cd-treated and control. Figure 7 shows the parameters of the comet cells included % DNA in tail and tail length.

Comet cells of the control of every treatment displayed a circle-like shape in which the whole nuclei and DNA were beautifully stained with the fluorescent color. The condition of Cd-treated cells indicated the presence of a clouded comet tail in which the % DNA in tail and tail length were increased significantly (*p* < 0.05) because of Cd-induced the oxidative damage to cells. In both of pre-treatment and co-treatment conditions, the RG and Se-RG treated cells significantly exhibited the reduction in the % DNA in tail and tail length compared to the Cd-treated group (negative control), and thus illustrated a DNA protective effect. The results on comet assay parameters of each treatment were correlated to the content of MDA production.

The damaging of DNA could be a subsequent effect on the production of high ROS and lipid radicals induced by Cd. The role of flavone glycosides as a natural antioxidant in the RG and Se-RG may influence the protective property by possibly up-regulating the level of the antioxidant defense system and abolishing oxidative DNA damage via the donation of electrons to reactive metabolites and rendering them inactive to prevent the interaction to the DNA. The experiment on the protective role of flavonoid compounds as an excellent radical scavenger to reduce the DNA damage in human blood lymphocyte is also consistent with this result [38]. Se-RG showed higher ability on the reduction in the tail length and % DNA in the tail of the comets compared to the RG. This indicated that Se in combination with the polyphenols could provide an extra protection and promote a protective role for the kidney cells. Se as the cofactor of various endogenous enzymes works in the antioxidant system could support the activity on the destructive of ROS. Fischer, et al. [39] suggested another possible role of Se, especially in the organic form, of protecting DNA damage via induction of p53 DNA repair pathway and transactivation of p53-regulated effector genes.

In the post-treatment condition, although the addition of the RG and Se-RG showed no significant effect on an improvement in cell viability of HEK293 cells, a minor role of DNA protection can visibly be seen. In the Cd-treated condition, most DNA in cells were broken down as indicated by the size of the comet head being obviously decreased from the control. DNA fragments were spread into the surrounding area as detected from the blurred green background. However, the addition of the extracts could not save the cell viability from Cd exposure, and the intensive black background remained to be observed. This might indicate a slight reduction in the number of DNA fragments in the surrounding area as both RG and Se-RG indicated a reduction in % DNA in the tail of comets compared to Cd-treated cells. Moreover, the addition of Se-RG indicated significant reduction in the tail length (*p* < 0.05), and thus showed better DNA protective properties.

## 4. Conclusions

In conclusion, our results showed the protective effect of RG and Se-RG in counteracting Cd-induced damage in HEK293 kidney cells during pre-treatment and co-treatment with Cd. The results could support the hypothesis that polyphenols in combination with Se compounds may help in the reduction of oxidative stress, lipid peroxidation rate, morphological impairments and DNA damage to kidney cells. Flavone glycosides as the major polyphenols in the extracts should contribute to these beneficial effects. Se-enrichment to ricegrass could promote additional benefits over typical ricegrass through the upregulation of the GPx enzyme. Se-RG should have potential to be produced and consumed as a functional food to protect the human body from Cd contamination.

## Figures and Tables

**Table 1:** The gradient condition of ultra-high-performance liquid chromatography–diode array absorbance detector–electron spray ionization–mass detector (UHPLC–DAD–ESI–MS).

Time (min)	% Solvent B
0.00–10.00	0.00–10.00
10.01–15.00	10.00
15.01–20.00	10.00–15.00
20.01–30.00	15.00–25.00
30.01–35.00	25.00
35.01–45.00	25.00–100.00

**Table 2:** The experimental treatment group on anti-cadmium toxicity and DNA protective properties.

	Control		Negative Control		Sample	
Time	24 h + 24 h		24 h + 24 h		24 h + 24 h	
Pre-incubation	Media	Media	Media	CdCl_2_ *	Extracts **	CdCl_2_
Co-incubation	Media	-	Media + CdCl_2_	-	Extracts + CdCl_2_	-
Post-incubation	Media	Media	CdCl_2_	Media	CdCl_2_	Extracts

* CdCl_2_ at CC_50_ level was used. ** Extracts from RG and Se-RG at 10,000 µg/mL were used.

**Table 3:** Tentative identification of phenolic compounds in ricegrass juice extract (RG) and Se-rich ricegrass juice extract (Se-RG) analyzed by UHPLC–ESI–MS.

Tentative Compounds	MW *	[M − H]^+^ (*m*/*z*)	References
Tricin	330	329	[22,23]
1-*O*-Sinapoyl-β-d-glucose	386	385	[22]
3-*O*-Feruloylquinic acid	368	367	[22,24]
Chrysoeriol arabinosyl arabinoside	564	563	[22,24]
Swertisin	446	445	[22]
Tricin-7-*O*-β-d-glucopyranoside	492	491	[25]

* MW: Molecular weight.

## References

[1] Tchounwou P.B., Yedjou C.G., Patlolla A.K., Sutton D.J., Luch A. (2012). Heavy metal toxicity and the environment. Molecular, Clinical and Environmental Toxicology, Experientia Supplementum.

[2] Elinder C.G., Järup L. (1996). Cadmium exposure and health risks: Recent findings. Ambio.

[3] Wang X.Q., Xu D., Lü M.K., Yuan D.R., Yin X., Zhang G.H., Xu S.X., Lu G.W., Song C.F., Guo S.Y. (2001). Crystal growth and physical properties of UV nonlinear optical crystal zinc cadmium thiocyanate, ZnCd(SCN)_4_. Chem. Phys. Lett..

[4] Järup L. (2003). Hazards of heavy metal contamination. Br. Med. Bull..

[5] Rahimzadeh M.R., Rahimzadeh M.R., Kazemi S., Moghadamnia A.A. (2017). Cadmium toxicity and treatment: An update. Casp. J. Intern. Med..

[6] Liu J., Qu W., Kadiiska M.B. (2009). Role of oxidative stress in cadmium toxicity and carcinogenesis. Toxicol. Appl. Pharmacol..

[7] Shaikh Z.A., Vu T.T., Zaman K. (1999). Oxidative stress as a mechanism of chronic cadmium-induced hepatotoxicity and renal toxicity and protection by antioxidants. Toxicol. Appl. Pharmacol..

[8] Nahak G., Suar M., Sahu R.K. (2014). Antioxidant Potential and Nutritional Values of Vegetables: A Review. Res. J. Med. Plant.

[9] Chomchan R., Siripongvutikorn S., Puttarak P., Rattanapon R. (2016). Investigation of phytochemical constituents, phenolic profiles and antioxidant activities of ricegrass juice compared to wheatgrass juice. Funct. Foods Health Dis..

[10] Sandbichler A.M., Höckner M. (2016). Cadmium protection strategies—A hidden trade-off?. Int. J. Mol. Sci..

[11] Mukherjee A., Sharma A. (1988). Effects of cadmium and selenium on cell division and chromosomal aberrations in *Allium sativum* L.. Water Air Soil Pollut..

[12] Chomchan R., Siripongvutikorn S., Puttarak P. (2017). Selenium bio-fortification: An alternative to improve phytochemicals and bioactivities of plant foods. Funct. Foods. Health Dis..

[13] Chomchan R., Siripongvutikorn S., Puttarak P., Rattanapon R. (2017). Influence of selenium bio-fortification on nutritional compositions, bioactive compounds content and anti-oxidative properties of young ricegrass (*Oryza sativa* L.). Funct. Food Health. Dis..

[14] Louis K.S., Siegel A.C., Stoddart M. (2011). Cell viability analysis using trypan blue: Manual and automated methods. Mammalian Cell Viability. Methods in Molecular Biology (Methods and Protocols).

[15] Du Y., Esfandi R., Willmore W.G., Tsopmo A. (2016). Antioxidant activity of oat proteins derived peptides in stressed hepatic HepG2 cells. Antioxidants.

[16] Bradford M.M. (1976). A rapid and sensitive method for the quantitation of microgram quantities of protein utilizing the principle of protein-dye binding. Anal. Biochem..

[17] Chen L., Yang X., Jiao H., Zhao B. (2002). Tea catechins protect against lead-induced cytotoxicity, lipid peroxidation, and membrane fluidity in HepG2 cells. Toxicol. Sci..

[18] Ercal N., Gurer-Orhan H., Aykin-Burns N. (2001). Toxic metals and oxidative stress part I: Mechanisms involved in metal-induced oxidative damage. Curr. Top. Med. Chem..

[19] Lindahl T., Barnes D. (2000). Repair of endogenous DNA damage. Cold Spring Harb. Symp. Quant. Biol..

[20] Singh N.P. (2000). Microgels for estimation of DNA strand breaks, DNA protein crosslinks and apoptosis. Mutat. Res..

[21] Besson E., Dellamonica G., Chopin J., Markham K.R., Kim M., Koh H.-S., Fukami H. (1985). C-glycosylflavones from *Oryza sativa*. Phytochemisty.

[22] Yang Z., Nakabayashi R., Okazaki Y., Mori T., Takamatsu S., Kitanaka S., Kikuchi J., Saito K. (2014). Toward better annotation in plant metabolomics: Isolation and structure elucidation of 36 specialized metabolites from *Oryza sativa* (rice) by using MS/MS and NMR analyses. Metabolomics.

[23] Kim J.H., Cheon Y.M., Kim B.G., Ahn J.H. (2008). Analysis of flavonoids and characterization of the *OsFNS* gene involved in flavone biosynthesis in rice. J. Plant Biol..

[24] Pourrut B., Pinelli E., Celiz Mendiola V., Silvestre J., Douay F. (2015). Recommendations for increasing alkaline comet assay reliability in plants. Mutagenesis.

[25] Henkler F., Brinkmann J., Luch A. (2010). The role of oxidative stress in carcinogenesis induced by metals and xenobiotics. Cancers.

[26] Langdon S.R., Mulgrew J., Paolini G.V., van Hoorn W.P. (2010). Predicting cytotoxicity from heterogeneous data sources with Bayesian learning. J. Cheminform..

[27] Johri N., Jacquillet G., Unwin R. (2010). Heavy metal poisoning: The effects of cadmium on the kidney. Biometals.

[28] Eneman J., Potts R., Osier M., Shukla G., Lee C., Chiu J., Hart B. (2000). Suppressed oxidant-induced apoptosis in cadmium adapted alveolar epithelial cells and its potential involvement in cadmium carcinogenesis. Toxicology.

[29] Ayala A., Muñoz M.F., Argüelles S. (2014). Lipid peroxidation: Production, metabolism, and signaling mechanisms of malondialdehyde and 4-hydroxy-2-nonenal. Oxid. Med. Cell. Longev..

[30] Cai Q., Rahn R.O., Zhang R. (1997). Dietary flavonoids, quercetin, luteolin and genistein, reduce oxidative DNA damage and lipid peroxidation and quench free radicals. Cancer Lett..

[31] Ding Y., Wang S.Y., Yang D.J., Chang M.H., Chen Y.C. (2015). Alleviative effects of litchi (*Litchi chinensis Sonn*.) flower on lipid peroxidation and protein degradation in emulsified pork meatballs. J. Food Drug Anal..

[32] Renugadevi J., Prabu S.M. (2010). Quercetin protects against oxidative stress-related renal dysfunction by cadmium in rats. Exp. Toxicol. Pathol..

[33] Choi J.H., Rhee I.K., Park K.Y., Park K.Y., Kim J.K., Rhee S.J. (2003). Action of green tea catechin on bone metabolic disorder in chronic cadmium-poisoned rats. Life Sci..

[34] El-Sharaky A., Newairy A., Badreldeen M., Eweda S., Sheweita S. (2007). Protective role of selenium against renal toxicity induced by cadmium in rats. Toxicology.

[35] Liu L., Yang B., Cheng Y., Lin H. (2015). Ameliorative effects of selenium on cadmium-induced oxidative stress and endoplasmic reticulum stress in the chicken kidney. Biol. Trace Elem. Res..

[36] Ognjanovic B., Markovic S., Pavlovic S., Zikic R., Stajn A., Saicic Z. (2008). Effect of chronic cadmium exposure on antioxidant defense system in some tissues of rats: Protective effect of selenium. Physiol. Res..

[37] Zhou Y.J., Zhang S.P., Liu C.W., Cai Y.Q. (2009). The protection of selenium on ROS mediated-apoptosis by mitochondria dysfunction in cadmium-induced LLC-PK1 cells. Toxicol. In Vitro.

[38] Devipriya N., Sudheer A.R., Srinivasan M., Menon V.P. (2008). Quercetin ameliorates gamma radiation-induced DNA damage and biochemical changes in human peripheral blood lymphocytes. Mutat. Res..

[39] Fischer J.L., Lancia J.K., Mathur A., Smith M.L. (2006). Selenium protection from DNA damage involves a Ref1/p53/Brca1 protein complex. Anticancer Res..

